# Polymicrobial Infection with Major Periodontal Pathogens Induced Periodontal Disease and Aortic Atherosclerosis in Hyperlipidemic ApoE^null^ Mice

**DOI:** 10.1371/journal.pone.0057178

**Published:** 2013-02-25

**Authors:** Mercedes F. Rivera, Ju-Youn Lee, Monika Aneja, Vishalkant Goswami, Liying Liu, Irina M. Velsko, Sasanka S. Chukkapalli, Indraneel Bhattacharyya, Hao Chen, Alexandra R. Lucas, Lakshmyya N. Kesavalu

**Affiliations:** 1 Department of Periodontology, College of Dentistry, University of Florida, Gainesville, Florida, United States of America; 2 Department of Oral Biology, College of Dentistry, University of Florida, Gainesville, Florida, United States of America; 3 Departments of Medicine and Molecular Genetics & Microbiology, Division of Cardiovascular Medicine, College of Medicine, University of Florida, Gainesville, Florida, United States of America; 4 Department of Oral Diagnostic Sciences, College of Dentistry, University of Florida, Gainesville, Florida, United States of America; 5 Department of Periodontology, School of Dentistry, Pusan National University, Yangsan, Gyeongsangnam-do, Korea; The University of Texas at San Antonio, United States of America

## Abstract

Periodontal disease (PD) and atherosclerosis are both polymicrobial and multifactorial and although observational studies supported the association, the causative relationship between these two diseases is not yet established. Polymicrobial infection-induced periodontal disease is postulated to accelerate atherosclerotic plaque growth by enhancing atherosclerotic risk factors of orally infected Apolipoprotein E deficient (ApoE^null^) mice. At 16 weeks of infection, samples of blood, mandible, maxilla, aorta, heart, spleen, and liver were collected, analyzed for bacterial genomic DNA, immune response, inflammation, alveolar bone loss, serum inflammatory marker, atherosclerosis risk factors, and aortic atherosclerosis. PCR analysis of polymicrobial-infected (*Porphyromonas gingivalis* [*P. gingivalis*], *Treponema denticola* [*T. denticola*], and *Tannerella forsythia* [*T. forsythia*]) mice resulted in detection of bacterial genomic DNA in oral plaque samples indicating colonization of the oral cavity by all three species. Fluorescent *in situ* hybridization detected *P. gingivalis* and *T. denticola* within gingival tissues of infected mice and morphometric analysis showed an increase in palatal alveolar bone loss (p<0.0001) and intrabony defects suggesting development of periodontal disease in this model. Polymicrobial-infected mice also showed an increase in aortic plaque area (p<0.05) with macrophage accumulation, enhanced serum amyloid A, and increased serum cholesterol and triglycerides. A systemic infection was indicated by the detection of bacterial genomic DNA in the aorta and liver of infected mice and elevated levels of bacterial specific IgG antibodies (p<0.0001). This study was a unique effort to understand the effects of a polymicrobial infection with *P. gingivalis, T. denticola* and *T. forsythia* on periodontal disease and associated atherosclerosis in ApoE^null^ mice.

## Introduction

The polymicrobial nature of periodontal disease (PD) promotes chronic inflammation and orchestrates a complex disease mechanism in which inflammation results in the destruction of the periodontium (alveolar bone, cementum, periodontal ligament, and gingiva). Periodontal disease is becoming better recognized as a risk for and contributing factor to multiple systemic diseases, including atherosclerotic vascular disease (ASVD), diabetes, rheumatoid arthritis, and Alzheimer’s disease [1], [2], [3], [4], [5], [6]. Recent systematic reviews and meta-analysis of observational studies to date support an association between PD and ASVD independent of known confounders, but a casual relationship is not yet established [1], [7]. There is also a significantly increased prevalence and incidence of coronary heart disease (CHD) in patients with periodontal disease indicating that PD independently predicts CHD [8].

The existence of polymicrobial consortium in periodontal disease has been established by previous studies [9], [10]. The bacterial species *Porphyromonas gingivalis* (*P. gingivalis*), *Treponema denticola* (*T. denticola*) and *Tannerella forsythia* (*T. forsythia*) are strongly implicated in development of periodontal disease, and together are known as the “red complex” [11]. These bacteria colonize the oral biofilm late in its development, by coaggregation with the help of bridging bacteria including *Fusobacterium nucleatum* (*F. nucleatum*) [12], [13], [14], [15].

Many studies demonstrated that periodontal disease-associated bacteria enter the blood stream during mastication, brushing and flossing teeth, and during dental procedures [16]. Frequent, recurrent transient bacteremia has the potential to produce a chronic insult to the vasculature and may contribute to the injury and inflammation that initiates the development of atherosclerosis [3], [5], [6], [17]. In addition, periodontal lesions are recognized as continually renewing reservoirs for the systemic spread of bacteria and viruses, and their associated antigens, cytokines, and other proinflammatory mediators [5], [6]. Bacterial genomic 16S rDNA from numerous oral and periodontal species, including *P. gingivalis*, *T. denticola*, *T. forsythia,* and *F. nucleatum* have been detected in human clinical atherosclerotic plaque lesions [18], [19], [20], [21]. Among the microorganisms detected in atherosclerotic vessels, *P. gingivalis* does not appear dominant, nor does it appear consistently, and it is rarely detected without the presence of other organisms [18], [22], [23], which highlights the importance of studying how polymicrobial infection influences atherosclerosis development.

Prior *in vivo* studies have been limited to monobacterial infection with *P. gingivalis* and/or ligature models which are not truly representative of the inherent polymicrobial periodontal disease process [24], [25], [26], [27], [28], [29], [30]. Many monoinfection studies with *P. gingivalis* in ApoE^null^ mice have documented genomic bacterial DNA in inflamed aortic tissue with atheroma in the aortic root as well as detecting macrophage, T cells, and Toll-like receptors 2 and 4 (TLR-2, TLR-4), proinflammatory cytokines such as interleukin-6 (IL-6) and vascular cell adhesion molecule-1 (VCAM-1) in the aorta [24], [25], [31], [32].

Because of interspecies interactions, polymicrobial infections have the potential to result in greater deleterious effects on local oral infections and systemic infection in ASVD [1]. Furthermore, a recent report indicated that bacteria, viruses, mycoplasma, and fungi are associated with ASVD development, demonstrating that the microbiology of the atherosclerotic plaque is complex [1]. Polymicrobial infections are believed to accelerate PD, but whether a combined polymicrobial infection with *P. gingivalis+T. denticola+T. forsythia* will induce enhanced PD, bacteremia, systemic inflammation, and simultaneously accelerate atherosclerosis is as yet unknown.

Previous polymicrobial PD models in rats documented that the synergism of this infectious consortium results in increased alveolar bone resorption when compared to monoinfection [33]. Thus, it is apparent that periodontal disease is always polymicrobial in nature and that a reductionistic approach, using only one bacteria, will never show the true picture of events found throughout the progression of disease [34]. Therefore, the aim of this study is to develop a mouse model of periodontal disease induced via an oral infection with a polymicrobial consortium resulting in measurable effects of microbial colonization, PD progression, and pathogen dissemination through the circulation and the potential risk for invasion of the vasculature and initiation of inflammatory atherosclerosis. The primary emphasis of this study was not designed to examine the ability of an individual organism to induce periodontitis and atherosclerosis but to focus on evaluating polymicrobial infection-induced oral and systemic effects.

## Materials and Methods

### Microbial Strains and Inocula

*P. gingivalis* FDC 381, *T. denticola* ATCC 35404, and *T. forsythia* ATCC 43037 were used in this study and were routinely cultured anaerobically at 37°C as described previously [33], [35]. Bacterial concentration was determined and cells were resuspended in reduced transport fluid (RTF) at 10^10^ cells *per* mL [35]. For topical oral polymicrobial infection, *P. gingivalis* was mixed with an equal quantity of *T. denticola* for 5 min; subsequently, *T. forsythia* was added to the culture tubes containing *P. gingivalis* and *T. denticola*, and cells were mixed thoroughly and allowed to interact for an additional 5 min. *P. gingivalis*, *T. denticola*, and *T. forsythia* were then mixed with an equal volume of 4% (w/v) sterile carboxymethylcellulose (CMC; Sigma-Aldrich, St. Louis, MO) in phosphate buffered saline (PBS), and this mixture was used for oral infection (5×10^9^ bacteria *per* mL) in ApoE^null^ mice as described previously [35].

### ApoE^null^ Mouse Infection and Oral Plaque Sampling

**Figure 1:**
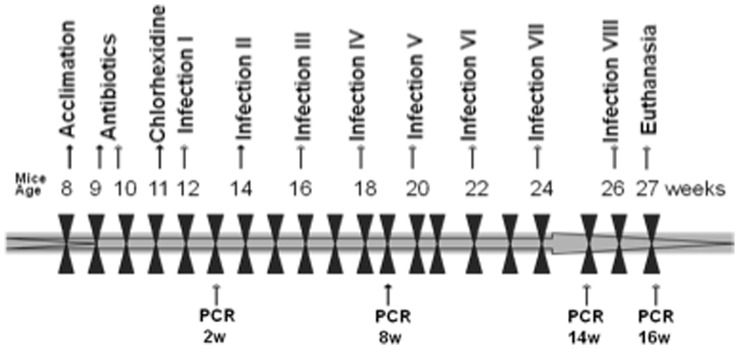
Schematic diagram. Schematic diagram of the experimental design illustrating the length of the study and highlighting important time points. Eight polymicrobial infections (Infection I through Infection VIII) are indicated at 12, 14, 16, 18, 20, 22, 24 and 26 weeks of age. Oral plaque samplings are indicated by PCR at week 2, 8, 14 and 16 following the initial infection. Blood and tissue specimen collection was performed at euthanasia following 16 weeks of infection.

The polymicrobial oral infection and sampling methodology were described previously [35] (Figure 1). Briefly, proatherogenic ApoE^null^ mice were used as a model for atherosclerosis [35], [36] and to examine the role of oral pathogen in induction of atherosclerosis [24], [25]. Male ApoE^−/−^ B6.129P2-*Apoe^tm1Unc^*/J mice, eight-weeks-old (The Jackson Laboratories, Bar Harbor, ME) were kept in groups and housed in microisolator plastic cages. Animals were fed standard chow and water *ad libitum,* and were randomly distributed into two groups; one for polymicrobial infection (n = 15) and one for sham-infection (n = 10). All mouse procedures were performed in accordance with the approved protocol guidelines by the IACUC of the University of Florida (IACUC Protocol # F173). ApoE^null^ mice were administered sulfamethoxazole (0.87 mg *per* mL) and trimethoprim (0.17 mg per ml) daily for 10 days in the drinking water [35] and the mouse oral cavity was rinsed with 0.12% chlorhexidine gluconate (Peridex: 3M ESPE Dental Products, St. Paul, MN) mouth rinse [33], [37] to inhibit endogenous microorganisms and to enhance subsequent colonization of human periodontal bacteria [33]. The polymicrobial inoculum (5×10^9^ combined bacteria per ml; 1×10^9^ cells in 0.2 ml per mouse; 3.3×10^8^ *P. gingivalis*, 3.3×10^8^ *T. denticola*, and 3.3×10^8^ *T. forsythia*) was administered topically to polymicrobial infection group (n = 15) for 4 consecutive days, every other week, for a total of 16 weeks to mimic chronic exposure during this period (Fig. 1). Control uninfected mice (n = 10) were inoculated with sterile 2% CMC only. Oral microbial samples were collected at 7 days postinfection [35]. In order to monitor the infection with minimal disruption of the biofilms, a total of 4 post-infection microbial samples (following weeks 2, 8, 14, and 16) were collected from all infected mice (Figure 1). The samples were collected by swabbing the oral cavity of the mice using a sterile veterinary cotton swab with a head width of 2.6 mm. The teeth and surrounding gingival tissue are swabbed and the cotton tip is immersed in 10∶1 TE buffer. The resuspended bacterial cells are used for DNA extraction and PCR.

All mice were monitored daily until euthanasia and mice appeared healthy throughout the experimental period. Following 16 weeks of polymicrobial infection, mice were euthanized and the blood, maxilla and mandibles, aorta, heart, spleen, liver, and kidneys were collected. Blood was collected, serum separated, and stored at −20°C for immunoglobulin G (IgG) antibody analysis [37] and serum cholesterol evaluation [25]. The mouse left maxillae and mandibular regions were resected from each mouse, autoclaved, and mechanically defleshed for evaluation of maxillary and mandibular alveolar bone loss by morphometric analysis [37]. The mouse right mandibular region was also resected from each mouse and immediately fixed in 10% neutral buffered formalin and decalcified with Immunocal (Decal Chemical Corporation, Tallman, NY) for 28 days at 4°C for histology and morphometric analysis [37], [38], [39].

### Detection of *P. gingivalis, T. denticola,* and *T. forsythia* Genomic DNA in Oral Plaque

DNA was isolated from mouse oral plaque samples using the Wizard Genomic DNA Purification Kit (Promega, Madison, WI) following manufacturer’s protocol. PCR was performed with a Bio-Rad thermal cycler using 16S rRNA gene species-specific oligonucleotide primers (*P. gingivalis*): 5′-TGTAGATGACTGATGGTGAAAACC-3′ (forward), 5′-ACGTCATCCCCACCTTCCTC-3′ (reverse); (*T. denticola)* TAATACCGAATGTGCTCATTTACAT-3′(forward), 5′-CTGCCATATCTCTATGTCATTGCTCTT-3′ (reverse); and (*T. forsythia*) 5′-AAAACAGGGGTTCCGCATGG-3′ (forward), 5′-TTCACCGCGGACTTAACAGC-3′ (reverse) [33], [37]. Genomic DNA extracted from these three strains served as positive controls and PCR performed with no template DNA served as negative control. PCR was performed in a 50 µl reaction mixture containing Phusion® High-Fidelity PCR Master Mix (New England Biolabs, Ipswich, MA), template DNA and 0.2 µM of oligonucleotide primers, using the following parameters: 1 cycle of initial denaturation was performed at 98°C for 30 seconds, 35 cycles of a denaturing step at 98°C for 10 seconds, a primer-annealing step at 52°C for 30 seconds and an extension step at 72°C for 30 seconds, a final extension cycle was performed at 72°C for 5 min. PCR products were separated by 1.5% agarose gel electrophoresis and the bands were visualized using a BioRad Gel Doc XR/Chemidoc Gel Documentation System (BioRad, CA, USA). Each PCR assay could detect at least 0.05 pg of DNA standard.

### Serum Antibody Analysis

Sera from infected mice (n = 15) at 16 weeks were used to determine immunoglobulin G (IgG) and IgM antibody concentrations against whole cells (formalin-killed) of *P. gingivalis, T. denticola,* and *T. forsythia* measured by an enzyme-linked immunosorbent assay (ELISA) [33], [37], [38], [39]. Briefly, whole *P. gingivalis, T. denticola,* and *T. forsythia* cells were treated overnight with 0.5% formalin in buffered saline (FK cells), washed, diluted to OD_600_ 0.3, and coated in wells of microtiter plates [33]. Diluted mice sera (1∶100 for IgG and 1∶20 for IgM) were reacted with the bacterial antigen for 2 h at room temperature. After washing, the secondary antibody goat anti-mouse IgG and IgM conjugated to alkaline phosphatase (1∶5000) (Bethyl Laboratories, Montgomery, TX) were added to the plates and the assay developed with *p*-nitrophenolphosphate (Sigma-Aldrich). The assay reactions were terminated by the addition of 3M NaOH and analyzed at OD_405_ using a Bio-Rad Microplate Reader. Mice serum antibody concentrations were assessed using a gravimetric standard curve that consisted of 8 mouse IgG and IgM concentrations (Sigma-Aldrich), which were coated onto wells, detected, and developed as described previously [38], [39].

### Morphometric Analysis of Periodontal Alveolar Bone Loss

The horizontal alveolar bone resorption (ABR) area and the presence of periodontal intrabony defects were measured by histomorphometry as described previously [37], . Briefly, the maxilla and mandible (n = 10−15) were immersed in 3% (vol/vol) hydrogen peroxide overnight after autoclaving and defleshing the maxillae and mandibles and stained in an aqueous solution of 0.1% methylene blue to delineate the cemento-enamel junction (CEJ) [37], [38], [39]. Digital images of both buccal and lingual root surfaces of all molar teeth were captured under a 10×stereo dissecting microscope (SteReo Discovery V8; Carl Zeiss Microimaging, Inc, Thornwood, NY), after superimposition of buccal and lingual cusps to ensure reproducibility and consistency. The line tool was used to measure the horizontal alveolar bone resorption from the CEJ to the alveolar bone crest (ABC). The surface perimeters of CEJ and ABC were traced using the calibrated line tool (AxioVision LE 29A software version 4.6.3.). Two blinded examiners performed all measurements twice at separate times. The means of the measurements were obtained for each of the two quadrants. Periodontal intrabony defects were detected under a 10×stereo dissecting microscope (SteReo Discovery V8) by an experienced periodontist (JL). The maxillae and mandibles were tilted and stabilized with dental wax to verify the presence of the intrabony defects in buccal and lingual surfaces. Only the presence or absence of intrabony defects was detected because the crevasses in the mouse jaw are too small to measure depth and width.

### Detection of Bacterial Genomic DNA in Internal Organs

Genomic DNA was isolated from heart, liver, spleen, abdominal aorta and thoracic aorta tissue samples using the Phenol:Chloroform method of extraction [36]. Tissue cells were lysed using a 1∶50 solution mixture of DNA Extraction Buffer (10 mM Tris-base, 0.1 M EDTA and 0.5% SDS) and Proteinase K Solution (Invitrogen, Carlsbad, CA) shaking at 600 rpm, 55°C, overnight. The supernatant was collected, Phenol:Chloroform:Isoamyl Alcohol (Invitrogen, Carlsbad, CA) was added and this mixture was allowed to shake at 300 rpm, room temperature, for 2 h. The supernatant was once again collected and this step was repeated for 2 additional hours. After spinning down, the top layer was collected, 100% cold ethanol and 10 M Ammonium Acetate was added and the mixture was stored at −20°C overnight. The next day the pellet was washed with 75% cold ethanol and dissolved in molecular grade water. PCR was performed as previously described for oral plaque samples.

### Morphometric Analysis of Aortic Atherosclerosis

The heart, aortic arch, thoracic aorta, and abdominal aorta were harvested from the mice after euthanasia. The aorta was cut into two equal parts, arch/thoracic (termed thoracic) and abdominal aortic lengths. The aortic root arising from the heart was also isolated as this is a site of accelerated plaque in ApoE^null^ mice [40]. The largest plaques are typically detected in the ascending aorta as it emerges from the heart at the level of the aortic valve. Each specimen was then cut into two sections, one for isolation of bacterial genomic DNA and the other for histology. Each section (half of the heart, aortic root, thoracic aorta, and abdominal aorta) were then fixed in 10% neutral buffered formalin, processed and paraffin embedded as previously described [36], [41], [42], [43]. Paraffin embedded samples were cut transversely into cross sections of 5 µm thick cryosections on the Leica EG 1160 Cryostat (Leica Microsystems Inc, Bannockburn, IL), two to three cross sections taken along the length of each segment. Sections were stained with Hematoxylin and Eosin (H&E) for morphometric analysis using an Olympus DP7 color video camera attached to an Olympus BX51 microscope (Olympus America, Center Valley, PA). The mean plaque area, lumen, and internal elastic lamina (IEL) area, intimal and medial thickness ratios, as well as immune cell counts were measured using the Image Pro system MC 6.0 software program (Olympus America, Center Valley, PA), with measurements adjusted to the microscopic objective. The mean total cross-sectional intimal plaque area and the mean intimal thickness normalized to the medial thickness (I/M) was calculated for each arterial section, the mean for measurements from each aorta was calculated for each mouse, and used for statistical analyses. Finally, the images were digitized and analyzed as previously described [36], [44], [45].

### Fluorescent *In Situ* Hybridization (FISH)

FISH allows the detection of bacterial species-specific ribosomal nucleic acid sequences in cells by binding to oligonucleotide probes to their complementary target sequences for *in situ* detection of bacteria [46]. *In situ* hybridization was performed on formalin-fixed paraffin-embedded infected right mandible tissue sections using Alexafluor-568 (Invitrogen, Carlsbad, CA) 3′-labeled oligonucleotide probes specific for *P. gingivalis* 5′-CAATACTCGTATCGCCCGTTATTC-3′ [47], *T. dentcola* 5′-CATGACTACCGTCATCAAAGAAGC-3′ [48], [49], [50] or *T. forsythia* 5′-CGTATCTCATTTTATTCCCCTGTA-3′ [47], [51] 16S rRNA. The emission maximum for the Alexafluor fluorescent dye is 578/603 nm, red-orange fluorescence. The protocol was modified accordingly [46]. Tissue sections on slides were deparaffinized in decreasing concentrations of xylene and ethanol: 100% xylene 6 min, 50–50 xylene-ethanol, 100% ethanol, 95% ethanol, 75% ethanol, 50% ethanol 3 min each, running water 5 min. Samples were blocked with Denhardt’s reagent (Fisher, Waltham, MA) and covered with hybridization solution (900 mM NaCl, 20 mM Tris-HCl pH 7.5, 0.01% SDS, 20% formamide) containing 5 µg/ml probe, and incubated for 3 h at 46°C. The probe was rinsed off and incubated in wash buffer (20 mM Tris-HCl, pH7.5, 5 mM EDTA, 0.01% SDS, 0.225 M NaCl), at 48°C for 25 min. Blocking buffer, hybridization buffer and wash buffer all contained protectRNA (Sigma-Aldrich) to protect bacterial RNA from degradation. Tissue sections were counter-stained with DAPI and mounted with mowiol. Stained slides dried overnight before being viewed under a fluorescence microscope. Images were acquired using a spinning disk confocal system connected to a Leica DMIRB microscope with a 63X oil immersion objective, equipped with a Photometrics cascade-cooled EMCCD camera, under the control of the open-source software package µManager (http://www.micro-manager.org/).

### Inflammatory Biomarker Serum Amyloid A (SAA)

Sera from polymicrobial-infected and sham-infected mice (n = 6) at 16 weeks were used to detect acute phase reactant SAA concentrations using the Mouse Serum Amyloid A ELISA kit (Kamiya Biomedical Company, Seattle, WA) [52]. The kit contained pre-coated plates containing bound anti-SAA antibody that was used for both calibrations and samples. All procedures were performed at room temperature. Diluted (1∶100) serum samples were added to pre-coated plate and incubated for 1 h. After washing the wells using washing solution from the kit, Enzyme-Antibody Conjugate was added and the plate was incubated for 30 min. After another wash TMB substrate solution from the kit was added and incubated for 10 min. The reaction was stopped using the stop solution contained in the kit. Absorbance was determined at OD_450_ using a Bio-Rad Microplate Reader. A second order polynomial calibration curve was made using the standards contained in the kit. The kit contained a mouse SAA calibrator of known concentration (10.5 µg/ml) and this was reconstituted and diluted to the suggested dilutions. The diluted samples of known concentrations were then included in the ELISA run along with our samples. The data was analyzed for statistical significance by Mann-Whitney t-test.

### Serum Cholesterol Analysis

Serum levels of total cholesterol, triglycerides, glucose, insulin, and serum creatinine were analyzed (Shands Medical Laboratories, Rocky Point Core Lab, Gainesville, FL) after euthanasia in mice infected with periodontal pathogens and sham-infected control [25]. Serum cholesterol levels were determined using the Roche Cholesterol liquid reagent assay on a Roche/Hitachi 917 analyzer (Roche Diagnostics, IN). Serum triglyceride levels were assessed using the Roche triglyceride assay with liquid triglycerides reagents on the Roche/Hitachi 917 and Roche/Hitachi 717 analyzers (Roche Diagnostics; IN). Similarly, glucose serum level was assessed using the Roche Glucose HK liquid assay on Roche/Hitachi 917 and 717 analyzers (Roche Diagnostics; IN). Serum insulin levels were assessed using the Roche Elecsys Insulin assay with the Enzymun-Test Insulin method (Roche Diagnostics; IN). The serum creatinine determination was performed using the Roche Creatinine plus assay with a creatinine HPLC method (Roche Diagnostics; IN).

### Histology of Gingival Inflammation

Histomorphologic analysis of the right mandible was examined for gingival inflammation according to previously established protocol [37]. Briefly, right mandibles of polymicrobial-infected (n = 15) and sham-infected (n = 10) mice were removed and fixed in 10% neutral buffered formalin for 24 h. The tissue was decalcified, embedded, sectioned and stained as described previously [38], [39]. The interproximal areas between the molars in each specimen were examined and images were taken at 20X magnification. Degree of inflammation, type of inflammatory cells, apical migration of junctional epithelium, and epithelial hyperplasia were recorded [37].

### Statistical Analysis

Antibody analysis and alveolar bone resorption data are presented in figures as means ± standard deviations (SD). Unpaired, two-tailed Student’s *t-*test was used to compare two independent groups. Simple regression analysis was performed to evaluate the association between bacterial DNA presence and aortic plaque formation. The serum amyloid A data was analyzed for statistical significance by Mann-Whitney t-test. For all statistical analysis, Prism for Windows, version 5.0 (GraphPad Software, San Diego, CA) or Statsview statistic package were used (*p*<0.05 considered significant).

## Results

### Monitoring Oral Polymicrobial Infection

Subsequent to treatment with sulfamethoxazole and trimethoprim, ApoE^null^ mice were monitored for the presence of human periodontal pathogens (*P. gingivalis, T. denticola and T. forsythia*) by PCR using bacterium-specific primers. Oral plaque samples were obtained from both pathogen infected and sham-infected mice, in the weeks following oral polymicrobial infections (weeks 2, 8, 14, and 16) and screened by PCR. This was done to confirm colonization/infection of the bacterial inocula. PCR analysis of samples collected post-infection at week 2 resulted in (n = 10, 0, 1) detection of infected mice positive for *P. gingivalis, T. denticola,* and *T. forsythia*, respectively (Table 1). PCR analysis following week 8 of infections also detected (n = 15, 15, 15) positive mice, with detected bacterial DNA at week 14 (n = 15, 13, 15) and at week 16 (n = 13, 13, 14) in infected mice positive for these three periodontal pathogens, respectively (Table 1). No sham-infected mice were positive for *P. gingivalis, T. denticola,* or *T. forsythia*, at any of the time points examined (Table 1).

**Table 1:** Distribution of ApoE^null^ mice oral microbial samples positive for bacterial genomic DNA by PCR.

	No. of oral microbial samples positive for bacterial genomic DNA											
	P. gingivalis				T. denticola				T. forsythia			
	2 wk^a^	8 wk	14 wk	16 wk	2 wk	8 wk	14 wk	16 wk	2 wk	8 wk	14 wk	16 wk
Infected^b^ n = 15	10	15	15	13	0	15	13	13	1	15	15	14
Control^c^ n = 10	–	–	–	–	–	–	–	–	–	–	–	–

a Indicate time points at which oral microbial samples were collected (2, 8, 14, and 16 weeks) following polymicrobial infection for determination of microbial colonization by species specific PCR analysis.

b Indicate mice were infected for 8 alternate weeks with *P. gingivalis*, *T. denticola*, and *T. forsythia*. Samples were analyzed using appropriate specific PCR primers with positive and negative controls.

c Oral microbial samples were collected from sham-infected control mice periodically and examined for *P. gingivalis*, *T. denticola*, and *T. forsythia* using bacteria specific primers and all mice were negative. - Indicate oral microbial samples negative by PCR analysis. *Pg*-indicate *P. gingivalis*; *Td*-indicate *T. denticola*; and *Tf*-indicate *T. forsythia*.

### Antibody Response to *P. gingivalis/T. denticola/T. forsythia*

**Figure 2:**
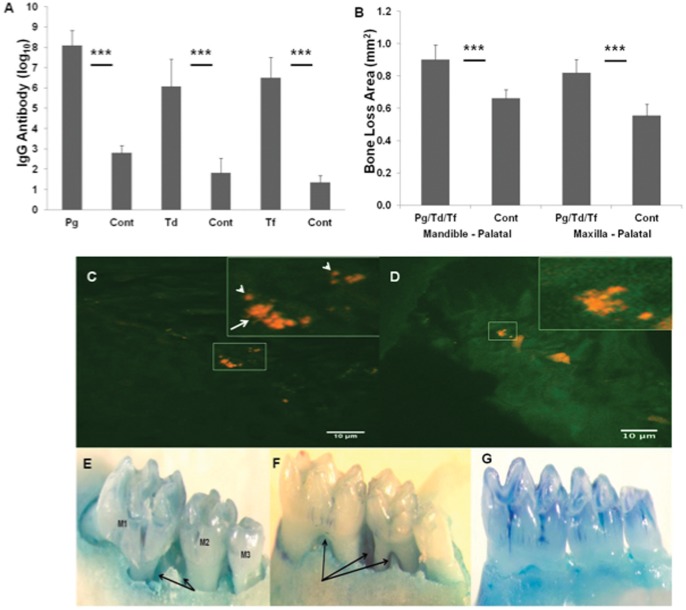
Periodontal disease parameters. (**A**) Serum IgG antibody levels from ApoE^null^ mice collected after 16 weeks of oral polymicrobial infection with *P. gingivalis*, *T. denticola,* and *T. forsythia* (n = 10−15). The graph shows the results for IgG antibody reactive with each of the three bacterial species. The bars indicate mean antibody levels in serum from mice infected with consortia or from controls. Significantly higher levels of IgG antibody were seen in polymicrobial-infected mice when compared to sham-infected controls. (**B**) Area of horizontal alveolar bone resorption in mandibular and maxillary palatal surfaces in ApoE^null^ mice. Each bar indicates the mean alveolar bone resorption for three molars in each quadrant of infected (n = 15) and sham-infected mice (n = 10). The vertical line denotes standard deviation from mean. *** Asterisks indicate significantly different (*P*<0.0001) than the sham-infected controls. The IgG antibody levels are expressed as log_10_. *Pg* indicates *P. gingivalis*; *Td* indicates *T. denticola*; *Tf* indicates *T. forsythia*; Cont indicates sham-infected control mice. *Pg*/*Td*/*Tf* indicates polymicrobial infection with *P. gingivalis*, *T. denticola* and *T. forsythia*. (**C, D**) Fluorescent *in situ* hybridization of formalin-fixed paraffin-embedded right mandible section from a polymicrobial-infected mouse using Alexafluor-568 labeled 16S RNA species specific (*P. gingivalis, T. dentcola* and *T. forsythia*) oligonucleotide probes. Red fluorescence indicates the presence of bacterial 16S RNA. The white arrow in the zoomed in square points to *T. dentcola* spirochete morphotype. The white arrow heads point to *P. gingivalis* coccobacilli morphotype. (**E, F**) Representative images of maxilla and mandible palatal surfaces showing extensive interproximal intrabony defects in mice infected with *Pg/Td/Tf*. M1 indicates first molar, M2 indicates second molar and M3 indicates third molar. Black arrows indicates intrabony defects. (**G**) Sham-infected mouse mandible-palatal showing no intrabony defects.

To provide additional confirmation of oral polymicrobial infection and to document a bacterial specific humoral response to polymicrobial infection, we evaluated pathogen-specific serum IgG and IgM levels against formalin-killed whole cells for *P. gingivalis, T. denticola,* and *T. forsythia* in mice sera from both polymicrobial-infected and sham-infected mice (Figure 2A). All mice in the polymicrobial-infected group for 16 weeks demonstrated significantly (*P*<0.0001) elevated IgG antibody to *P. gingivalis, T. forsythia,* and *T. denticola* compared to the levels in sham-infected control mice. However, anti-*P. gingivalis* IgG antibody levels were higher than anti-*T. forsythia* and anti-*T. denticola* IgG antibody levels. Among the three pathogens, *P. gingivalis* and *T. forsythia* induced levels of serum IgG antibody in polymicrobial infected mice approximately 100,000-fold compared to the levels found in the sham-infected mice. Similarly, *T. denticola* induced levels of serum IgG antibody in polymicrobial infected mice approximately 10,000-fold compared to the levels of sham-infected mice (Figure 2A). None of the polymicrobial-infected mice induced IgM antibody to *P. gingivalis/T. forsythia/T. denticola* during 16 weeks of infection (data not shown).

### Alveolar Bone Resorption and Intrabony Defects

The progression of PD resulting from polymicrobial infection with *P. gingivalis/T. denticola/T. forsythia* was examined by measuring the effects of polymicrobial infection on ABR. The mandible and maxilla of polymicrobial-infected and sham-infected ApoE^null^ mice were collected at necropsy and morphometric analysis was performed in order to confirm ABR (Figure 2B). The findings demonstrated significantly higher (*P*<0.0001) palatal horizontal ABR in both the mandible and maxilla of polymicrobial infected mice when compared to sham-infected mice. The measured mean alveolar bone loss area found in the mandible of polymicrobial-infected mice was 0.90 mm^2^ compared to only 0.66 mm^2^ in sham-infected mice. The mean bone loss area measured in the maxilla of polymicrobial-infected mice resulted in 0.82 mm^2^ compared to only 0.55 mm^2^ in sham-infected mice (Figure 2B, *P*<0.0001). The presence or absence of intrabony defects was also examined (Figure 2E, F and G). We found that 53.33% of the total surface of both the mandible and maxilla of polymicrobial infected mice contained intrabony defects compared to only 22.5% of the teeth surfaces of sham-infected mice (Table 2).

**Table 2:** Polymicrobial infection-induced periodontal intrabony defects in ApoE^null^ mouse.

Jaw/Surfaces	Poly-microbial Infection	Sham-infected Control
Maxilla	58/90* ^a^ (64.44%)** ^b^	19/60 (31.67%)
Mandible	38/90 (42.22%)	8/60 (13.33%)
Buccal	38/90 (42.22%)	11/60 (18.33%)
Palatal	58/90 (64.44%)	16/60 (26.67%)
Total	96/180 (53.33%)	27/120 (22.5%)

* ^a^The frequency was calculated by tooth surfaces containing periodontal intrabony defects out of total tooth surfaces. Calculation of total tooth surface: polymicrobial-infected group has 90 tooth surfaces [15 mice×3 molars×2 sides (buccal, palatal)] and control has 60 (10 mice×3 molars×2 sides).

** ^b^Numbers in the parenthesis indicate the percentage of tooth surface with periodontal intrabony defects.

### Microbial Systemic Invasion and Detection of Genomic DNA from Aorta, Heart, Liver, and Spleen

The heart, aorta, liver, and spleen were harvested post euthanasia and examined for the presence of *P. gingivalis, T. denticola,* and *T. forsythia* genomic DNA via PCR with bacterium-specific primers. Phenol:chloroform DNA extraction was performed on one half of each mouse heart for both polymicrobial-infected and sham-infected mice. PCR analysis revealed 1 out of 15 polymicrobial-infected mice were positive for the existence of *P. gingivalis* genomic DNA in the heart (Table 3). PCR analysis of thoracic and abdominal aortic samples revealed the presence of a polymicrobial infection for both areas of the aorta in several of the polymicrobial-infected mice. In the thoracic and abdominal aorta 9 out of 15 and 10 out of 15 mice were found to contain *P. gingivalis* genomic DNA, respectively. *T. denticola* genomic DNA was found in the thoracic aorta of 12 out of 15 polymicrobial-infected mice. Similarly, analysis of the thoracic and abdominal aorta resulted in a positive finding of *T*. *forsythia* DNA in 12 out of 15 and 9 out of 15 mice, respectively. No bacterial genomic DNA was detected in any of the organs sampled from the sham-infected mice (Table 3).

**Table 3:** Detection of *P. gingivalis, T. denticola, T. forsythia* genomic DNA in ApoE^null^ mouse tissue.

	Thoracic Aorta	Abdominal Aorta	Heart	Liver	Spleen
	*Pg/Td/Tf*	*Pg/Td/Tf*	*Pg/Td/Tf*	*Pg/Td/Tf*	*Pg/Td/Tf*
Infected n = 15	9/12/12	10/0/9	1/0/0	7/1/5	0/1/0
Control n = 10	0/0/0	0/0/0	0/0/0	0/0/0	0/0/0

Post-euthanasia the thoracic aorta, abdominal aorta, heart, liver and spleen were harvested in liquid nitrogen. In order to assess systemic infection with *P. gingivalis, T. denticola* and/or *T. forsythia,* total DNA from each perspective organ was isolated and examined for bacterial genomic DNA by PCR using species specific primers. The numbers indicated with forward slash correspond to the number of mice positive for *Pg/Td/Tf* respectively.

### Fluorescent *In situ* Hybridization

All three organisms were detected in infected mouse gingival tissues by fluorescent *in situ* hybridization with oligonucleotide probes to *P. gingivalis*, *T. denticola*, and *T*. *forsythia*. Clusters of both *P. gingivalis* and *T. denticola* are seen (Figure 2C and D). Individual cells of *P. gingivalis* can be seen (white arrow heads, Figure 2C) as well as individual spiral shaped *T. denticola* (white arrow Figure 2C). *T. forsythia* could not be identified in gingival tissues. The presence of these pathogens clearly demonstrates that they invaded gingival epithelium and possibly entered circulation and thus complements the genomic DNA data results (Table 3).

### Histomorphometric Analysis of Atherosclerotic Plaque

**Figure 3:**
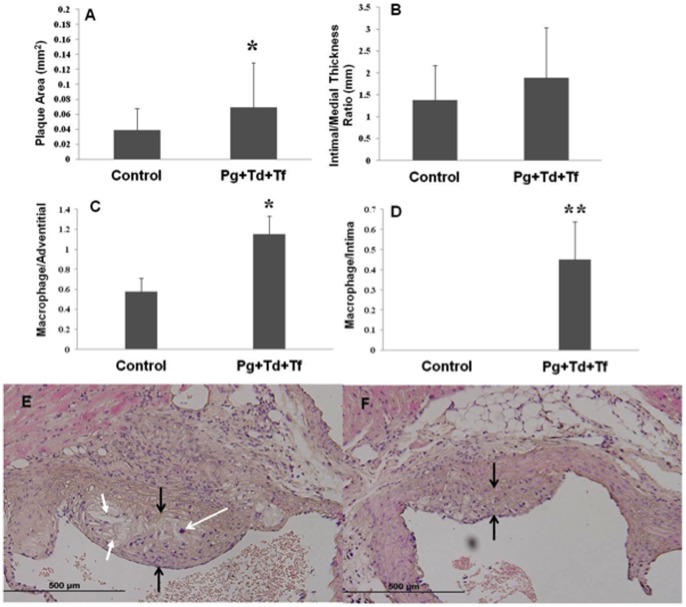
Atherosclerotic vascular disease parameters. (**A**) The total aortic plaque area was measured and an increase in plaque area (mm^2^) was found for polymicrobial-infected ApoE^null^ mice when compared to sham-infected control mice (*P*<0.05). (**B**) Intimal/medial thickness ratios were determined and an increase was observed in the polymicrobial-infected mice compared to sham-infected mice. Polymicrobial infection-induced macrophage infiltration in ApoE^null^ mice. 4 µm thick paraffin embedded sections of the aortic arch were cut and stained with H&E; (**C**) Number of macrophage per total adventitial area. (**D**) Number of macrophage per total intimal area. Control indicates sham-infected ApoE^null^ mice and *Pg*+*Td*+*Tf* indicate polymicrobial-infected mice. An increase in macrophages was seen in both the adventitial and intimal layers in the polymicrobial-infected mice when compared to the sham-infected mice. Histologic evaluation of 4 µm thick paraffin embedded aortic arch sections stained using H? plaque area is flanked with black arrows, macrophage infiltration is indicated using white arrow with open head and cholesterol crystals are indicated using white arrows with filled heads. (**E**) Sham-infected control mice tissue (Magnification 40×); (**F**) *Pg, Td,* and *Tf* infection mice tissue (Magnification 40×) containing larger plaque area, increased macrophage infiltration and increased presence of cholesterol crystals, suggesting polymicrobial infection-induced aortic plaque and aortic wall thickness in ApoE^null^ mice.

Atherosclerotic plaque was measured by two experienced researchers (AL, LL) blinded to the experimental groups. Histologic cross sections of the ascending aorta demonstrated increased inflammatory cell invasion in the intimal and adventitial layers of polymicrobial-infected mice and associated increased plaque growth when compared to sham-infected mice. The presence of increased plaque area was proportional to an increase in the number of bacterial species found. The presence of polymicrobial infection produced a trend in increased plaque area (Figure 3A). Intimal/medial thickness ratios indicated a similar trend but were not significant with increased intimal thickness ratios in polymicrobial infections (Figure 3B). The number of invading mononuclear cells in the adventitial (Figure 3C) and intimal (Figure 3D) layers was similarly increased in mice with polymicrobial infections. With polymicrobial infection, mean ascending aortic plaque area was increased with invading foam cell macrophage (p<0.05) (Figure 3E) when compared to sham-infected controls (Figure 3F).

### Polymicrobial Infection and the Effect of Atherosclerosis Risk Factors

We determined whether established atherosclerosis risk factors were present in order to document whether periodontal pathogens are able to modulate risk factors during 16 weeks of oral infection (Table 4). The serum total cholesterol level for infected mice (n = 13) was significantly higher than sham-infected mice (*P*<0.05). Similarly, serum total triglyceride levels were also significantly (*P*<0.001) higher in polymicrobial-infected (n = 15) when compared to sham-infected control mice. The serum glucose level for polymicrobial-infected mice (n = 5) was lower than sham-infected mice but did not reach significance. However, there are no observed differences in insulin or serum creatinine levels between polymicrobial-infected and sham-infected mice (n = 5) (Table 4).

**Table 4:** Determination of polymicrobial oral Infection-induced risk factors for atherosclerosis.

Risk Factors	Poly-microbial Infection	Sham-infected Control
Total cholesterol, mg *per* dL^a^	508.8±19.27*	442.4±13.94
Total triglyceride, mg *per* dL^b^	208.7±9.765**	155.0±8.747
Glucose, mg *per* dL^c^	181.6±16.93	207.2±24.52
Insulin, ng *per* mL^c^	<0.5	<0.5
Creatinine, mg *per* dL^c^	0.1–<0.1	<0.1

Values are mean±SD;

* indicates *P*<0.05;

** indicates *P*<0.001;

a n = 13 mice per group;

b n = 15 mice per group;

c n = 5 mice per group.

### Polymicrobial Infection-induced Inflammatory Biomarker SAA

**Figure 4:**
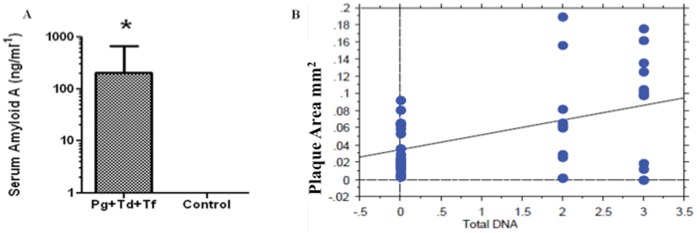
Serum Amyloid A and Linear regression plot. (**A**) ELISA analysis of antibody levels to Serum Amyloid A (SAA). *Pg/Td/Tf* infected mice showed approximately 110-fold increase in SAA levels compared to sham-infected mice (*P*<0.05). (**B**) A simple regression analysis was performed using the STATview software of the aortic plaque area in mm^2^ versus the number of bacterial species found by PCR, using species specific primers, in the aorta of polymicrobial infected mice. The simple regression plot demonstrates a positive correlation between the number of detected bacterial species and plaque size in the aorta (R^2^ = 0.192, P<0.005).

Since Serum Amyloid A (SAA) is one of the major acute phase proteins mainly associated with high density lipoproteins, a quantitative determination of SAA was performed in the polymicrobial-infected mice. The results showed a significant (*P*<0.05) increase (110-fold) in SAA production in polymicrobial-infected mice when compared to the sham-infected mice (Figure 4A).

### Correlation between Number of Bacterial Species Detected and Atherosclerotic Plaque Area

Regression analysis was performed to assess the association between the presence of polymicrobial species and the progression of atherosclerotic plaque. The total number of species detected by PCR, using species specific primers, (0-controls with no bacterial species found, 1-single bacterial species found, 2-two species found, or 3-three species found) was plotted versus the total aortic plaque area as measured in the aorta for each mouse (Figure 4B). A linear correlation between the number of total bacterial species detected in the aortic tissues and an increase in atherosclerotic plaque area was found (R^2^ = 0.192, P<0.005) (Figure 4B).

### Histology of Gingival Inflammation

Minimal differences were observed in the degree of inflammation, type of inflammatory cells, apical migration of junctional epithelium, and epithelial hyperplasia in right mandible of polymicrobial-infected and sham-infected mice (Data not shown).

## Discussion

Observational studies to date support an association between PD and ASVD independent of known confounders [1], [2], [3], [4], [5], [6]. Recent mechanistic *in vivo* studies clearly demonstrated the plausibility of a direct link between *P. gingivalis* monoinfections and atherogenesis in an ApoE^null^ mouse model and have documented biological pathways in induction of inflammatory atherosclerosis [24], [53]. However, human PD is a chronic infection that is exclusively initiated by complex subgingival biofilm including *P. gingivalis*, *T. denticola*, *T*. *forsythia*, *F. nucleatum*, and *P. intermedia*. Moreover, genomic DNA from nine periodontal bacteria and three viruses has been detected in inflammatory ASVD lesions, suggesting that the true nature of atherosclerotic lesions is of a polymicrobial nature. Prior monoinfection studies will not have direct relevance to the polymicrobial nature of periodontitis or serve as a precise model to examine the link between periodontitis and atherosclerosis induction. Thus, several periodontal pathogens as co-infection model are more appropriate indices to examine the role of their ability to induce vascular inflammation and ASVD. Although PD subgingival biofilms are more complex and polymicrobial, we infected with three well-characterized pathogens to examine their synergistic ability to induce periodontitis, dissemination, vascular inflammation, and initial stages of atherosclerosis. Furthermore, the primary emphasis of this study was to focus on evaluating polymicrobial infection-induced oral and systemic effects.

Successful oral colonization of mice with *P. gingivalis/T. denticola/T*. *forsythia* is a critical event in pathogenicity. Polymicrobial colonization in the ApoE^null^ mice oral cavity is consistent with our previous findings in the rat model of polymicrobial PD [33]. These three pathogens as a polymicrobial infection also induced greater ABR and more intrabony defects. Moreover, the alveolar bone crests showed rough and irregular margins which indicates that periodontitis was in progression. These results also provide direct evidence that these three pathogens colonized in the mice oral cavities and induced ABR similar to our previous observations in rats [33].

The polymicrobial oral infection elicited highest profiles for serum IgG antibodies in ApoE^null^ mice. Among the three pathogens, *P. gingivalis* infection elicited the highest levels of IgG antibody followed by *T. forsythia* while the levels of *T. denticola* antibodies were slightly lower than *T. forsythia.* These clearly indicate robust colonization of these pathogens and an induction of humoral immune response in infected ApoE^null^ mice. Furthermore, ApoE^null^ mice IgG antibody levels induced by these pathogens are significantly higher than rats [33] indicating higher colonization capacity of the individual pathogen in the polymicrobial consortium. Since previous *in vivo* studies were published with *P. gingivalis* in a monoinfection models using different infection protocols [24], [25], [26], we could not compare our polymicrobial infection immune response profiles, ABR, and development of atherosclerosis to previous monoinfection data.

Presence of genomic DNA of *P. gingivalis/T. denticola/T. forsythia* in internal organs such as thoracic and abdominal aorta and liver clearly indicate *P. gingivalis/T. denticola/T. forsythia* gained access to systemic circulation and to the aorta (bacteremic episodes) from gingival tissues and may have induced vascular wall lesions and promoted the formation of early atheromatous plaque in the aortic arch. Simple regression analysis found a positive correlation (p<0.05) between the number of species detected by genomic DNA of bacteria detected in the aorta and plaque size in thoracic aorta. The linear correlation derived from the presence of multiple species of bacteria and increased aortic plaque size pointed towards the cooperativity existing in the polymicrobial consortium towards initiation of pathogenesis, but defining the degree of cooperation was not the objective of our current study. Enhanced serum acute phase reactant SAA, a biomarker of inflammation [54], [55] demonstrated the role of periodontal pathogens in CVD. In addition, alteration of total cholesterol and triglycerides demonstrate the pathogen’s ability to modulate atherosclerosis risk factors [25]. Furthermore, detection of highly specific molecular signals with pathogen specific oligonucleotide probes provide strong evidence that these pathogens primarily invaded gingival tissues and entered systemic circulation and thereby initiated events inducing atherosclerosis.

In summary, our findings clearly showed (i) polymicrobial colonization/infection in the oral cavity of mice, (ii) specific serum IgG antibody response, (iii) increased ABR and intrabony defects, (iv) in* vivo* invasion in gingival tissues by FISH, (v) *P. gingivalis/T. denticola/T. forsythia* genomic DNA in aorta, (vi) induction of serum inflammatory marker SAA, (vii) alteration of serum cholesterol and triglycerides, (viii) induction of atherosclerotic plaque in aortic root, and (ix) the creation of a polymicrobial periodontal disease model in ApoE^null^ mice. This polymicrobial mouse model system serves as a better platform to further study the effects of putative periodontal pathogens in systemic diseases including atherosclerosis, rheumatoid arthritis, Alzheimer’s disease, and diabetes. Therefore, this study attempts to provide a newer experimental model to evaluate the cascade of events in polymicrobial consortium as well as it adds strong evidence for the involvement of major periodontal pathogens in CVD.
